# Anti-angiogenic properties of rapamycin on human retinal pericytes in an in vitro model of neovascular AMD via inhibition of the mTOR pathway

**DOI:** 10.1186/s12886-022-02334-w

**Published:** 2022-03-25

**Authors:** Ben Asani, Jakob Siedlecki, Christian Wertheimer, Raffael Liegl, Armin Wolf, Andreas Ohlmann, Siegfried Priglinger, Claudia Priglinger

**Affiliations:** 1 Department of Ophthalmology, Ludwig-Maximilians-University, Mathildenstrasse 8, 80336 Munich, Germany; 2 Department of Ophthalmology, University Clinic Ulm, Ulm, Germany

**Keywords:** Age related macular degeneration, Choroidal neovascularization, VEGF, PDGF, Rapamycin, Sirolimus, mTOR

## Abstract

**Purpose:**

Choroidal neovascularizations (CNV) are partially stabilized through a coverage of pericytes leading to a partial anti-VEGF resistence. Drugs licensed for neovascular AMD (nAMD) do not take this mechanical and growth factor-driven CNV stability into account. The purpose of this work was to see if inhibiting the mammalian target of rapamycin (mTOR) may successfully block angiogenic cellular pathways in primary human retinal pericytes in an in vitro model of nAMD.

**Methods:**

The mTOR inhibitor rapamycin was used to treat human retinal pericytes (HRP) at doses ranging from 0.005 to 15 g/ml. A modified metabolism-based XTT-Assay was used to assess toxicity and anti-proliferative effects. A scratch wound experiment showed the effects on migration. On Cultrex basement membrane gels, the influence of rapamycin on the development of endothelial cell capillary-like structures by human umbilical vein vascular endothelial cells (HUVEC) in the absence and presence of pericytes was investigated.

**Results:**

Rapamycin showed no signs of toxicity within its range of solubility. The drug showed dose dependent anti-proliferative activity and inhibited migration into the scratch wound. Endothelial cell tube formation in a HUVEC monoculture was effectively inhibited at 45%. A co-culture of HUVEC with pericytes on Cultrex induced endothelial tube stabilization but was disrupted by the addition of rapamycin leading to degradation of 94% of the tubes.

**Conclusions:**

Rapamycin allows for an efficient modulation of aspects of angiogenesis in pericytes via mTOR-modulation in vitro. Further studies are needed to elucidate whether rapamycin may have an impact on CNV in nAMD in vivo.

## Introduction

Current treatment strategies for neovascular AMD almost exclusively focus on vascular endothelial growth factor (VEGF) as their pharmacological target [1]. Offering a monotherapy only, all anti-VEGF substances approved nowadays have to be reapplied into the vitreous body regularly, often monthly [2, 3]. Moreover, long-term follow-up of patients under anti-VEGF therapy has revealed that in contrast to the visual gains reported after two years, mean visual acuity declines severly after five to seven years [4, 5]. Furthermore, 19.7% - 36.6% had still evidence of active exudation after one year of anti-VEGF therapy [6].

Many additional strategies to improve CNV management beyond anti-VEGF have been proposed [7]. In CNV, anti-VEGF targets endothelial cells, but not cells of the vascular wall [8]. It has frequently been hypothesized that the cells of the vascular wall, mainly pericytes, mediate partial anti-VEGF resistance [9–13]. Pericytes are VEGF-independent and rely on platelet-derived growth factor (PDGF) for survival and recruitment to sites of angiogenesis [14–17]. Exploiting this concept, a recent clinical phase 2b trial had demonstrated a beneficial effect of intravitreal anti-PDGF application on nAMD. However, the consecutive large clinical phase 3 trials simultaneously antagonizing VEGF and PDGF finally failed to show superior visual acuity in comparison to anti-VEGF monotherapy [10]. Inclusion criteria were slightly different from the phase 2b trial, which may have caused some bias. Nevertheless, since the phase 3 trials had failed to reach the primary endpoint, the combination therapy as a new option for treatment of nAMD was abandoned.

Thus, there is still an urgent need for alternative approaches targeting angiogenesis beyond VEGF alone as a plethora of *in vitro* and animal models is providing evidence that the combined inhibition of endothelial cells and pericytes exhibits stronger anti-neovascular properties than sole endothelial cell-targeted anti-VEGF therapy [9, 13, 18], other potential pathways to inhibit pericyte stabilization of CNV under anti-VEGF therapy are of great interest.

The mammalian target of rapamycin (mTOR) signaling pathway is a major regulator of cell growth and proliferation. When deregulated, it is associated with several different diseases including cancer and diabetes [19]. The drug Rapamycin as an inhibitor of the mTOR pathway is nowadays used in the treatment on a number of diseases including but not limited to lymphangioleiomyomatosis or as an immunosuppressant after a kidney transplant [20, 21].

Its function is mainly mediated through binding with the 12-kDa FK506-binding protein (FKBP12) and amplifying its effect as a direct inhibitor of mTOR. This leads to less activity of important upstream regulators such as the serine/threonine kinase S6K1 and PI3-Kinase signaling as an essential mediator of cellular growth and proliferation [19, 22–24].

In *in vivo* tumor models, inhibition of mTOR was found to reduce angiogenesis and microvascular density [25, 26]. In the eye, inhibition of the mTOR pathway has frequently been described as a new strategy to manage neovascularization in a variety of ocular diseases [27]. The following study was therefore performed to evaluate mTOR-inhibition as an alternative approach to PDGF-antagonism to target angiogenesis in retinal pericytes and endothelial cells *in vitro* and enhance the current management of CNV in nAMD.

## Materials & methods

### Rapamycin

Rapamycin powder (Sigma, St. Louis, MO, USA) was dissolved in 100% dimethyl sulfoxide (Sigma, St. Louis, MO, USA). The desired concentration of rapamycin was achieved through dilution in ready-to-use pericyte (Cell Systems, Seattle, USA) or endothelial cell growth medium (PromoCell, Heidelberg, Germany) with a final concentration of 0.1% DMSO for in vitro studies.

### Cell culture

Cultures of human retinal pericytes (HRP) were obtained from Cell Systems (Seattle, USA) and cultured along the manufacturer’s instructions in its respective growth medium with the included antibiotic (Bac-Off®) and growth factor-mix (CultureBoost™). All experiments were conducted on passages 3-7, performed as duplicates and repeated at least three times on different days.

Cultures of human umbilical vein endothelial cells (HUVEC) were obtained from PromoCell (Heidelberg, Germany) and cultured according to manufacturer’s instructions in its respective serum-free endothelial growth medium with the included growth supplement mix. All experiments were conducted on passages 3-7, performed as duplicates and repeated at least three times on different days.

### Cellular viability

HUVEC and pericytes were brought to confluence on 12 well Nunc multidishes (Thermo Scientific, Waltham, MA, USA), kept in growth-factor reduced medium for 24h to stop proliferation and treated with vehicle (DMSO; control) or increasing concentrations of rapamycin at 0.005, 0.05, 0.5, 1, 2.5, 5, 7.5, 10, 12.5 and 15 μg/ml, respectively. After 72 hours of treatment, the XTT-Assay was performed as described below. Differences in absorption compared to the untreated control were interpreted as differences in metabolic activity and cell count due to potential toxicity of rapamycin. All experiments were conducted on passages 3-7, performed as duplicates and repeated at least three times on different days.

### Cellular proliferation

HUVEC and pericytes were seeded on 12 well Nunc multidishes at about 10-15% of confluence (3x10^3^ cells/cm^2^). After attachment, cells in their log phase of proliferation were exposed to vehicle or rapamycin as described above. After 72 hours of treatment, an XTT-Assay was performed as described. Differences in absorption compared to the untreated control were interpreted as differences in cell count due to antiproliferative effects of rapamycin. All experiments were conducted on passages 3-7, performed as duplicates and repeated at least three times on different days.

### XTT dye reduction assay

The XTT-Assay was used as previously described by Scudiero [28] and modified for ocular cell culture by Spitzer [29] and Wertheimer [30]. The assay is based on the reduction of the tetrazolium reagent XTT ((2,3-Bis-(2-Methoxy-4-Nitro-5-Sulfophenyl)-2H-Tetrazolium-5-Carboxanilide) into an aqueous-soluble formazan in viable cells, enhanced by the addition of phenazine methosulfate (PMS).

After washing with PBS, 350 μl of XTT reagent, supplemented with 10 μl of 5 mM PMS, were added to each well. After incubation under standard cell culture conditions for one hour, optical density was measured at 450 nm in an ELISA reader (Spectramax 190; Molecular Devices, Sunnyvale, CA, USA). The results are expressed as mean percentage of optic density relative to controls representing 100%. All experiments were conducted on passages 3-7, performed as duplicates and repeated at least three times on different days.

### Scratch-induced migration assay

HRP migration was observed using an in vitro scratch assay as previously described by Liang et al. with a few modifications [31]. Cells were grown on 12 well Nunc multidishes and after reaching confluency, a linear scratch wound was applied to the cell monolayer using a 10 μl pipette tip. The wound was photographed using an inverted phase-contrast microscope with a digital camera (Leica Microsystems GmbH, Solms, Germany), and the exact location of photography was marked on the multidish with a felt tip. The cells were then treated with vehicle or increasing rapamycin concentrations of 0.005, 0.05, 0.5 and 5 μg/ml, respectively, and incubated under standard cell culture conditions. After 24 hours, the scratch wounds were photographed again at exactly the same, pre-documented location. Total wound closure (distance between the edge of the wound directly after injury and after 24 h) was assessed with the LAS area measuring tool (Leica, Solms, Germany). The results are expressed as percentage of reduction in wound closure distance with the untreated controls representing 100%. All experiments were conducted on passages 3-7, performed as duplicates and repeated at least three times on different days.

### Tube formation: HUVEC monoculture

Tube formation was observed as described previously [13] on Cultrex© (Trevigen, Gaithersburg, MD, USA), a gel secreted by the murine Engelbreth–Holm–Swarm (EHS)-tumor. The gel, also known as Matrigel, solidifies at 37 °C, forming a basement membrane rich in pro-angiogenic factors on top of which endothelial cells can form tubular complexes. Twenty-four hours before use, Cultrex was thawed on ice in a refrigerator at 4 °C. 50 μl Cultrex per well were placed into a 96-well plate, and solidification was induced in an incubator at 37 °C for 30 minutes. HUVEC were passaged to 1.5 × 10^5^ cells/μl and treated with 0.005, 0.05, 0.5 and 5 μg/ml rapamycin. A cell suspension of 100 μl (1.5 × 10^4^ cells) was gently added to each well on top of the solidified gel. After incubation at 37°C for 6 h, three images of each well were taken with an inverted phase contrast microscope and a digital camera (Leica, Solms, Germany). Total tube length was evaluated with help of the LAS distance tool (Leica, Solms, Germany) to quantify the length of the vascular system. Results are expressed as percentage of total tube length compared to the untreated control representing 100%. All experiments were conducted on passages 3-7, performed as duplicates and repeated at least three times on different days.

### Tube-formation: HUVEC/HRP co-culture

After preparing Cultrex as described above, HUVEC were passaged to 1.5 x 10^5^ cells/μl and HRP to 7.5 x 10^3^ cells/μl. Onto the first third of the wells, solely 100 μl of HUVEC suspension were added to create HUVEC monocultures. Then, HUVEC and HRP suspension were mixed to seed a HUVEC-HRP coculture on the remaining two thirds of wells as previously described by Stratman et al [32]. After incubation at standard cell culture conditions for 6 hours, three pictures of each well were taken to document the results of tube formation. Immediately afterwards, the co-cultures were treated with concentrations of rapamycin at 0.005, 0.05, 0.5 and 5 μg/ml. After incubation at standard cell culture conditions for another 24 hours, three pictures per well were taken again to document the remains of the tube structures seen after the first 6 hours. Total tube length was evaluated, and results expressed as percentage of the change in total tube length with the control representing 100% as done above for the HUVEC monoculture. All experiments were conducted on passages 3-7, performed as duplicates and repeated at least three times on different days.

### Statistical analysis

Statistical analysis was performed in SPSS 23.0 (SPSS Inc., Chicago, IL, USA). Comparisons between multiple groups were performed by ANOVA with the LSD post hoc test. *p*<0.05 was considered statistically significant with a 95% confidence interval. Graphs were plotted in Microsoft Excel showing the standard deviation.

## Results

### HRP and HUVEC cell viability

**Fig. 1:**
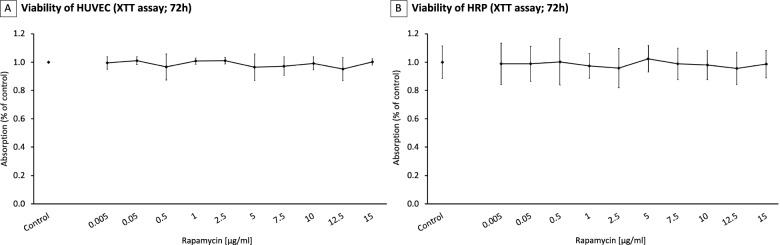
Toxicity of Rapamycin on HUVEC (**A**) and Human retinal pericytes (**B**). No toxicity on both HRP and HUVEC could be detected within the whole range of solubility

To exclude any toxic effects of rapamycin on cultured human umbilical vein endothelial cells (HUVEC) and human retinal pericytes (HRP), viability of the cells was investigated after exposure to increasing concentrations of rapamycin as a function of metabolic activity as determined by the XTT-assay. For this purpose, cells were maintained on growth factor depleted conditions. In both, HUVEC and HRP, no statistically significant decrease of metabolic activity could be observed for all tested concentrations ranging from 0.005 to 15 μg/ml (*p*>0.05 for all concentrations, Fig. 1).

### Rapamycin inhibits proliferation of HRP

**Fig. 2:**
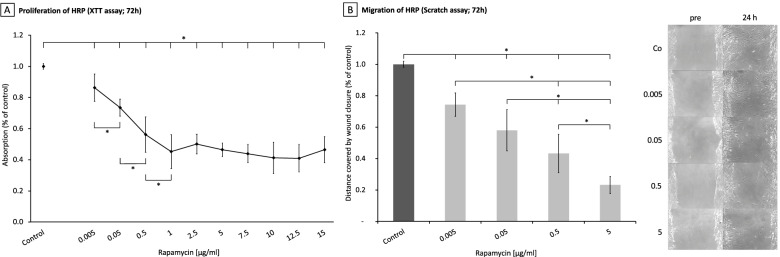
Effects of Rapamycin on proliferation (**A**) and migration (**B**) in pericytes A. Rapamycin significantly reduced proliferation in pericytes starting from 0.005μg/ml with a half maximal inhibitory concentration of 423 ng/ml. B. For all concentrations tested, Rapamycin dose-dependently reduced pericyte migration into the scratch wound after 24 hours (Co – Rapa 0.005: *p*=0.006; MD: 25.7; 95 % CI: 9.2 – 42.2; Co – Rapa 0.05: *p*<0.0001; MD 42.0; 95 % CI: 25.5 – 58.4; Co – Rapa 0.5: *p*<0.0001; MD 56.7; 95 % CI: 40.2 – 73.2; Co – Rapa 5: *p*<0.0001; MD 76.8; 95 % CI: 60.3-93.3). The control wound was densely covered with HRP after 24 hours in contrast to the highest used concentration of 5μg/ml where only little migration was present (*right*)

When added to cultured HRP in their log phase of proliferative activity rapamycin inhibited proliferation in HRP in a dose-dependent manner at all concentrations tested, ranging from 0.005 up to 15 μg/ml (*p*<0.05 for all concentrations). Dose-dependent effects were observed from 0.005 up to 1 μg/ml, where inhibition of proliferation reached a plateau with a maximal reduction of proliferative activity by 60%. The half maximal inhibitory concentration (IC_50_) was 423 ng/ml (Fig. 2).

### Influence of rapamycin on migration of HRP

The effects of rapamycin on migration of HRP were evaluated in a scratch assay and determined as percentage of the distance covered by the cells after 24 hours compared to the distance covered by the control cells, which was defined as 100% allowing for easy interpretation (distance between the wound gaps at 0h – distance at 24h = 100%). All tested rapamycin concentrations of 0.005, 0.05, 0.5 and 5 μg/ml significantly reduced the mean difference of wound closure by 25.7, 42.0, 56.7 and 76.8 %, respectively (Fig. 2 and Table 1). The inhibitory activity was dose dependant and was observed between 0.005 and 0.5 μg/ml, 0.005 and 5 μg/ml, 0.05μg/ml and 5 μg/ml as well as 0.5 and 5 μg/ml (Table 2).

**Table 1:** Effects of rapamycin on pericyte migration compared to untreated control

	*P* Value	Mean Deviation	95% CI
Co – rapa 0.005	0.006	25.7	9.2 – 42.2
Co – rapa 0.05	<0.0001	42.0	25.5 – 58.4
Co – rapa 0.5	<0.0001	56.7	40.2 - 73.2
Co- rapa 5	<0.0001	76.8	60.3 – 93.3

**Table 2:** Dose dependent effects of rapamycin on pericyte migration

	*P* Value	Mean Deviation	95% CI
rapa 0.005 – rapa 0.5	0.002	31.0	14.6 – 47.5
rapa 0.005 – rapa 5	<0.0001	51.1	34.7 – 67.6
rapa 0.05 – rapa 5	0.001	34.8	18.3 – 51.3
rapa 0.5 – rapa 5	0.022	20.1	3.6 – 36.6

### Effect of rapamycin on tube formation in a HUVEC monoculture

**Fig. 3:**
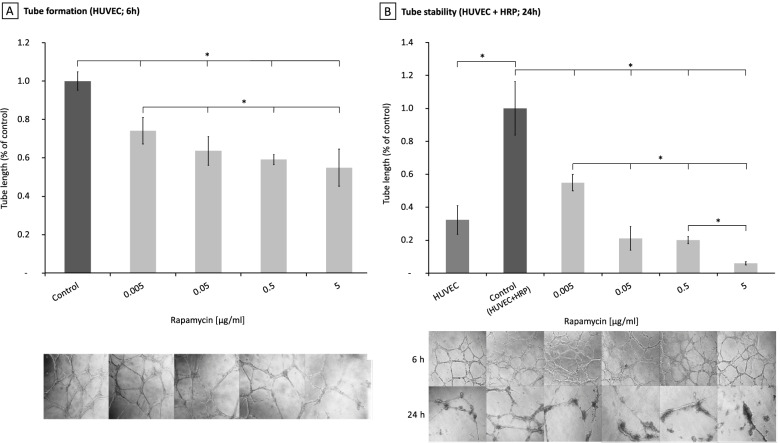
Effects of Rapamycin on tube formation in a HUVEC monoculture (**A**) compared to a HUVEC/HRP co-culture (**B**). Tube formation after 6 hours is significantly reduced under Rapamycin compared to the untreated control leading to a decrease down to 74.0%, 63.6%, 59.1% and 54.9% respectively (Co – Rapa 0.005: *p*=0.001; MD: 26.0; 95 % CI: 13.8 – 38.2; Co – Rapa 0.05: *p*<0.0001; MD 36.4; 95 % CI: 24.2 – 48.6; Co – Rapa 0.5: *p*<0.0001; MD 40.9; 95 % CI: 28.7 – 53.1; Co – Rapa 5: *p*<0.0001; MD 45.1; 95 % CI: 33.0-57.3). However, HUVEC monoculture tubes destabilize also without treatment after 30 hours while still being steady after the same time frame by adding pericytes and thus creating a co-culture comparable to the in vivo condition. B: Adding Rapamycin to the co culture tubes seems to disrupt endothelial tube stabilization (Co – Rapa 0.005: *p*<0.0001; MD: 45.1; 95 % CI: 30.2 – 59.9; Co – Rapa 0.05: *p*<0.0001; MD 78.9; 95 % CI: 64.1 – 93.8; Co – Rapa 0.5: *p*<0.0001; MD 80.0; 95 % CI: 65.1 – 94.8; Co – Rapa 5: *p*<0.0001; MD 94.0; 95 % CI: 79.1-108.9)

On regular Cultrex© all tested concentrations of rapamycin (0.005, 0.05, 0.5 and 5 μg/ml) led to a statistically significant decrease in the tube length by 26.0, 36.4, 40.9 and 45.1 %, respectively, relative to the untreated control (Fig. 3; Table 3). A dose dependent effect was observed between 0.005, 0.5 and 5 μg/ml (rapa 0.005 – rapa 0.5: *p*=0.021; MD 15.0; 95 % CI: 2.8 - 27.1; rapa 0.005 – rapa 5: *p*=0.006; MD 19.2; 95% CI: 7.0 – 31.4).

**Table 3:** Effects of rapamycin on HUVEC tube formation compared to the untreated control

	*P* Value	Mean Deviation	95% CI
Co – rapa 0.005	0.001	26.0	13.8 – 36.2
Co – rapa 0.05	<0.0001	36.4	24.2 – 48.6
Co – rapa 0.5	<0.0001	40.9	28.7 – 53.1
Co – rapa 5	<0.0001	44.1	33.0 – 57.3

### Rapamycin leads to a disruption of tube stability in a HRP and HUVEC co-culture

In vivo, endothelial tubes are sheathed and thereby stabilized by pericytes. In order to create a comparable condition in vitro, we established a co-culture of HUVEC and HRP.

Addition of HRP to the HUVEC monoculture led to a statistically significant increase in tube stability. The HUVEC monoculture showed 67.7 % more tube degradation after 30 hours when compared to the co-culture of HUVEC and HRP (HUVEC/HRP – HUVEC: *p*<0.0001; MD 67.7; 95 % CI: 52.9 – 82.6). All rapamycin concentrations tested (0.005, 0.05, 0.5 and 5 μg/ml) led to a statistically significant decrease of total tube length by 45.1, 78.9, 80.0 and 94.0 %, respectively, relative to the untreated co-culture control (Fig. 3, Table 4). A dose dependent effect was observed from 0.005 to 5 μg/ml (Rapa 0.005 – Rapa 0.5: *p*<0.0001; MD 49.0; 95 % CI: 34.1 – 63.8).

**Table 4:** Effects of rapamycin on tube stability in a co-culture tube formation

	*P* Value	Mean Deviation	95% CI
Co – rapa 0.005	0.001	26.0	13.8 – 36.2
Co – rapa 0.05	<0.0001	36.4	24.2 – 48.6
Co – rapa 0.5	<0.0001	40.9	28.7 – 53.1
Co – rapa 5	<0.0001	44.1	79.1 – 108.9

## Discussion

The pharmacological inhibition of VEGF has revolutionized the treatment of nAMD. However, many patient needs, mainly the long-term preservation of visual acuity and an alleviation of the current treatment burden, are still unmet [2, 4, 5]. These present limitations of anti-VEGF are largely attributable to insufficient CNV management, and the fact that cells of the vascular wall, mainly pericytes, and their promotion of vessel maturation and stabilization are unaffected by anti-VEGF as nowadays standard of care [9–13].

The goal of the present study was to examine the anti-angiogenic effects of rapamycin in an in vitro model of nAMD. Using well established models of cellular mechanisms involved in angiogenesis [12, 13, 31], we were able to show that the mTOR inhibitor rapamycin successfully suppresses endothelial cell tube formation comparable to anti-VEGF agents.

This is in line with previous findings showing that rapamycin can interfere with VEGF signaling [33], mainly interacting with VEGF-A driven early angiogenesis by modulation of the S6 tyrosine-kinase [34, 35]. Furthermore, as shown in lymphatic endothelial cells tube formation is reduced by rapamycin via downregulation of VEGF receptor 3 [36]. In our hands, rapamycin led to a significant dose-dependent reduction of total tube length in endothelial cell monocultures. Although not investigated in this work, this suggests that rapamycin might also interfere with VEGF receptor 2 which has been shown to be the main mediator of tubulogenesis in blood endothelial cells [37, 38]. This subject clearly awaits further investigation.

Liu et al. previously have shown that inhibiting mTOR can reduce proliferation and migration not only in endothelial cells, but also in pericytes in breast cancer models in mice [25]. Our study provides further evidence that rapamycin can also be used to modulate retinal pericyte growth in CNV angiogenesis by targeting the pathological vessel maturation and therefore add to the effect of anti-VEGF on the endothelial cell. Mechanistically, pericytes are thought to mediate a partial anti-VEGF resistance by conferring vascular stabilization via physical contact, VEGF and the Ang1/Tie2 system [16, 39, 40]. In our study, rapamycin dose-dependently reduced pericyte proliferation and migration and interfered with the pericyte stabilization of endothelial tubes, inducing vascular regression even in endothelial cell tube structures which had undergone pericyte coverage in a cellular co-culture. However, it is important to note that these are in vitro results and that other protocols on performing co-culture assays exist. Stahl et al. for example used two different layers of collagen gels for each cell line to investigate the effects of rapamycin on a co-culture system of RPE and endothelial cells [41]. For our specific purpose, the tubes had to be composed of both HUVEC and HRP, so we mixed both cell lines into one suspension and then placed it on the Matrigel. This allowed to form a tubular structure as a unit of both cell types which presumably mimics the actual situation in vivo. Albeit unlikely, the results might have had a different outcome using other protocols for the co-culture.

However, these findings reflect the wide efficacy of rapamycin on pericytes at two crucial time points in vessel maturation. First, as shown by Benjamin et al. [42], pericyte recruitment and vessel coverage lag behind primary endothelial cell driven angiogenesis, opening a plasticity window for vascular remodeling. By the addition of rapamycin to conventional anti-VEGF therapy, pericyte migration and proliferation to sites of nascent CNV might be limited, thus reducing the amount of pericyte coverage of maturing endothelial tubes. Secondly, already matured, pericyte covered endothelial tubes were significantly more vulnerable when rapamycin was added to the coculture. In a clinical context, this might allow for CNV regression if a combination therapy with conventional anti-VEGF inhibitors is applied [16]. In nAMD, VEGF-independent parts of CNV due to pericyte coverage might be forced back into VEGF-dependency by rapamycin-induced pericyte ablation.

From a clinical perspective, an induction of vessel regression is of high importance. Studies monitoring anti-VEGF treatment with optical coherence tomography angiography (OCT-A) have shown that CNVs react to the therapy by decreases in size and perfusion, but show persistence in their voluminous large vessel trunks and regrow upon sinking anti-VEGF levels [43, 44]. Phung et al. have demonstrated that pathological angiogenesis is induced and sustained by Akt activation, causing increased blood vessel size and ongoing edema due to higher vascular permeability [45]. Apart from its effects on the pericyte, rapamycin was suggested to reverse these changes by working as an inhibitor of endothelial Akt signaling, inducing vessel normalization of physiological segments [45]. Moreover, mature CNV segments forced into pericyte ablation by rapamycin might show delayed regrowth, and thus prolonged treatment intervals.

In a previous study we have shown that pericytes may contribute to subretinal fibrosis in nAMD [12]. This was recently confirmed by findings showing that pericytes activated by laser photocoagulation infiltrated the subretinal space in mice models, increasing the expressions of fibrogenic molecules, making them a potential therapy target for subretinal fibrosis [46]. In this context, pericyte inhibition using rapamycin might also modulate fibrotic responses of CNV generated in the macula, a frequent cause of serious vision loss in nAMD [47].

In conclusion, our *in vitro* data spurs further *in vitro* and *in vivo* studies on rapamycin as an antiangiogenic treatment for nAMD targeting the pericyte. As shown by recent phase 3 trials of rapamycin for the treatment of uveitis [48], intravitreal delivery of this promising substance can already be considered as safe. We believe that further investigation of this pathway, especially if the substance is to be delivered as a monotherapy or in combination with other anti-VEGF agents, could be a key in advancing the treatment options for neovascular AMD.

## Data Availability

The datasets used and/or analysed during the current study are available from the corresponding author on reasonable request due to ongoing further analysis.
